# Epidemiology and social factors of malaria in the Yanomami Indigenous Territory: a critical review of the challenges of control and elimination

**DOI:** 10.1590/0074-02760260033

**Published:** 2026-10-09

**Authors:** Djane Clarys Baia-da-Silva, Alexander Vargas, Edília Sâmela Freitas Santos, Karolina Jeaneth Solórzano Chavarría, Cláudio Tadeu Daniel-Ribeiro, Wuelton Marcelo Monteiro

**Affiliations:** 1 Fundação Oswaldo Cruz-Fiocruz, Instituto Leônidas e Maria Deane, Manaus, AM, Brasil; 2 Universidade Nilton Lins, Manaus, AM, Brasil; 3 Ministério da Saúde, Secretaria de Vigilância em Saúde e Ambiente, Brasília, DF, Brasil; 4 Fundação de Medicina Tropical Doutor Heitor Vieira Dourado, Manaus, AM, Brasil; 5 Universidade do Estado do Amazonas, Manaus, AM, Brasil; 6 Fundação Oswaldo Cruz-Fiocruz, Instituto Oswaldo Cruz, Laboratório de Pesquisa em Malária, Rio de Janeiro, RJ, Brasil; 7 Fundação Oswaldo Cruz-Fiocruz, Instituto Oswaldo Cruz, Centro de Pesquisa, Diagnóstico e Treinamento da Malária, Rio de Janeiro, RJ, Brasil; 8 Fundação Oswaldo Cruz-Fiocruz, Instituto Nacional de Ciência e Tecnologia em Imunomodulação, Rio de Janeiro, RJ, Brasil

**Keywords:** Amazon region, disease control, Indigenous health, gold mining, malaria, Yanomami

## Abstract

Malaria in the Yanomami Indigenous Territory (YIT) constitutes a serious public health concern and has been hyperendemic since 1987, following the arrival of illegal gold miners in the area. Disease transmission has steadily increased since 2013, particularly among children. It escalated into a crisis in 2019, driven by the intensification of illegal mining and environmental, sociocultural, and political factors, and reached approximately 33,000 cases in 2024. Although the declaration of a Public Health Emergency in 2023 led to advancements in surveillance and diagnosis, the incidence of cases remains elevated. Nevertheless, there was a remarkable reduction in malaria-related mortality, evidenced by a 42% decrease in deaths and a 47% decline in the case-fatality rate, even in the context of persistently high notification rates. Barriers to control include limited access to remote communities, ecological degradation that favours vector proliferation, a high prevalence of submicroscopic infections, and sociocultural constraints. Expanded diagnostic capacity and a reinforced workforce are essential but insufficient without addressing the underlying structural causes. This mini-review summarises and critically discusses the epidemiological trends of malaria in YIT, as well as the social, environmental, cultural, and operational factors that sustain transmission.

Malaria is a serious public health problem worldwide and in Brazil, where 139,884 cases were recorded in the Amazon region in 2023, accounting for 99.98% of all cases in the country.^(1)^ In the same year, malaria cases in Indigenous areas saw a significant increase, surpassing the number of cases in rural areas, something that had not occurred since 2003.^(1)^ Interpreting this increase demands caution. Rather than reflecting the onset of a sudden epidemic, the data more likely point to a steady, ongoing intensification of malaria transmission within the Yanomami Indigenous Territory (YIT). Part of the observed increase may be explained by improvements in surveillance efforts, expanded diagnostic access, and, consequently, more thorough case documentation, even as control operations remain disrupted and barriers to healthcare persist, particularly in isolated, difficult-to-reach locations.^(2)^

Several underlying structural and contextual factors further sustain the local transmission. Geographic isolation, the semi-nomadic movement typical of many Indigenous groups, and chronically limited health service availability not only facilitate the continued spread of *Plasmodium* spp., but also make swift diagnosis and treatment more difficult; these challenges are especially acute for the Yanomami population. Recurring intrusions by illegal miners, along with their environmental impact, have also played a significant role in driving up malaria transmission, as well as morbidity and mortality rates.

This mini-review aims to describe the epidemiological landscape in YIT and to analyse the social, cultural, environmental, and operational factors that sustain malaria transmission. Furthermore, it aims to identify the challenges and limitations to controlling and eliminating the disease, while considering the impact of illegal mining and structural vulnerabilities.

This narrative mini-review was based on secondary data obtained from peer-reviewed scientific articles, official surveillance databases, epidemiological bulletins, and institutional reports from the Brazilian Ministry of Health (MoH). The choice of sources was informed by their relevance to understanding malaria transmission dynamics, control strategies, and specific contextual factors of YIT. A systematic search protocol was not adopted, which may introduce some selection bias. Nevertheless, the aim was to select reliable references aligned with the analysed context to promote a comprehensive and well-founded interpretation of the available evidence.

## CHARACTERISATION OF THE YANOMAMI POPULATION

The Yanomami population's contact with wider society is relatively recent, dating back to the 1940s, although some groups remain isolated, while others demonstrate varying degrees of contact with society at large.^(3-6)^ An estimated 33,948 Yanomami live in 428 communities in the states of Roraima and Amazonas, northern Brazil, in a territory covering approximately 9.6 million hectares.^(7)^ In Brazil, the Indigenous Territory is inhabited by eight distinct ethnic groups organised into 37 Indigenous health subunits (pole bases). These subunits are distributed across the municipalities of Amajari (RR), Alto Alegre (RR), Mucajaí (RR), Iracema (RR), Caracaraí (RR), Barcelos (AM), Santa Isabel do Rio Negro (AM), and São Gabriel da Cachoeira (AM).^(8)^

The Yanomami are hunters, gatherers, and farmers (especially of bananas, cassava, sugarcane, corn, and tobacco).^(4,9,10)^ They are semi-nomadic and move around the territory for a variety of reasons, including hunting and management of natural resources and/or cultivating fields, divisions of groups for political or family reasons, visits to strengthen ties and exchanges between groups and/or conflict, participation in ceremonies and rituals,^(6,11,12,13)^ and they are currently at risk of infection with *Plasmodium* spp.^(4,8,14,15,16,17)^

The Yanomami population has endured decades of intense pressure stemming from mineral exploitation in their territory. In recent years, the pace of mining activity has intensified further, amid changes in environmental and Indigenous policies. Adding to this situation is the persistence of obstacles to access to healthcare and basic services, which amplify territorial and sanitary vulnerability. Together with the current political instability in neighbouring Venezuela, these factors contributed to one of the worst public health emergencies associated with Indigenous peoples in Brazil in recent years.^(18,19,20)^ Given the living and health conditions of the population in the territory, on January 20, 2023, the MoH declared a Public Health Emergency of National Importance (PHENI), aiming at the immediate intensification of health assistance for people living in the YIT, as well as establishing the Public Health Emergency Operations Centre (COE-Yanomami) as a national effort to address the crisis and coordinate the management of the emergency response.^(21,22)^

## MALARIA AMONG THE YANOMAMI POPULATION: HISTORICAL AND EPIDEMIOLOGICAL ASPECTS

Malaria currently represents one of the most critical public health threats in the Yanomami territory, characterised by increasing incidence and profound socio-epidemiological vulnerability. The disease was previously rare in the most peripheral villages, with no autochthonous transmission reported in the central areas of the territory until 1987.^(4,15)^ However, after the Amazon Radar Project revealed the region's high mineral potential in 1975, an intense migratory flow began, with estimates suggesting that approximately 40,000 prospectors entered the territory in search of riches.^(4)^ This disorganised population movement, combined with multiple factors such as precarious housing conditions, risky behaviours, climatic and hydrological variations, and the inappropriate use of medication, created a favourable scenario for the proliferation of malaria, substantially intensifying the epidemiological vulnerability of the area and exposing the population to a serious health risk.

A report on the evolution of the morbidity profiles of the Yanomami Indigenous people treated at the Indigenous Health House (CASAI) in Boa Vista, Roraima, showed that malaria was the leading cause of illness, with an approximately 500% increase in malaria cases, and the leading cause among the 114 deaths recorded between 1987 and 1989.^(15)^ Severe anaemia was among the leading manifestations of severe malaria.^(5)^ In the YIT, anaemia is prevalent; although multifactorial in origin, it may be partly attributable to recurrent episodes of the disease.^(5,23)^

In 1990, the federal government created the Yanomami Emergency Health Care Plan (PEASY) to address the severe living conditions in the region, including epidemics and malnutrition. The plan focused on managing health crises and conducting a population census. The most important actions were the active search for new malaria cases and the on-site examination and treatment of all residents within the villages to ensure adherence to treatment and to reduce drug resistance.^(4)^

The Yanomami Special Indigenous Health District (DSEI-Y), established in 1992, unified health services in the region, with a focus on basic care within communities and the provision of referrals for more complex cases. This structure, combined with the work of Urihi Yanomami Health between 1999 and 2004, was crucial in reducing infant mortality by 76% and malaria incidence by 84%, effectively eliminating the most severe cases of *Plasmodium falciparum* malaria in some regions.^(4)^

Between 2010 and 2017, the incidence of malaria increased by an average of 9.5% per year across a total mining area of 1.23 km². In the following period, from 2018 to 2023, a significant expansion of the mined area was observed, accompanied by a sharp jump in the annual growth rate of the disease, which reached 30.2%. This percentage represents more than three times the previous rate, translating into a marked acceleration in malaria transmission.^(8)^

In 2022, there was a reduction in reported cases, which may have been related to disruptions in access to health services, reduced diagnostic capacity, and the impact on the reliability of surveillance data, thus increasing the risk of underreporting. Changes in political and institutional priorities may have directly influenced the allocation of resources, the implementation of territorial protection initiatives, and the continuity of malaria control efforts, and should be considered to justify the reduction in cases that year. Some projections suggest that underreporting of malaria cases in 2022 may have reached rates as high as 83%. This was based on models presented by Castro et al.^(8)^ and on results from integrating epidemiological data and environmental indicators, such as the expansion of mining areas. However, it is crucial to interpret this estimate with caution, as it relies on inferences about the dynamics of disease transmission rather than on data derived from the active and direct detection of cases.^(8)^ Cases peaked at approximately 33,000 in 2024. Models indicate that more than 100,000 cases could have been avoided between 2018 and 2023 if the mined area had not expanded.^(8)^ This increase was predominantly driven by *Plasmodium vivax* infections but was accompanied by a concerning rise in *P. falciparum* malaria, which also reached a higher case count in 2024 and would actually point to an increase in transmission.

Over time, the number of reported malaria cases has increased significantly in YIT, primarily due to *P. vivax*, with a notable increase in 2024 during the PHENI (Figure), despite workforce expansion, increased diagnostic testing (rapid diagnostic testing), and improved treatment availability. During the PHENI, control efforts included prioritising active case search, mass examination of symptomatic and asymptomatic individuals, the introduction of tafenoquine as a new therapy for the treatment of uncomplicated *vivax* malaria, the use of artesunate plus mefloquine (ASMQ) as a first-line drug for the treatment of *falciparum* malaria, and the expansion of vector control activities with specialised teams. Endemic disease control agents and entomologists focused on mitigating breeding sites, such as mining craters, by implementing sanitation measures and conducting indoor residual spraying.^(24,25)^

**Figure:**
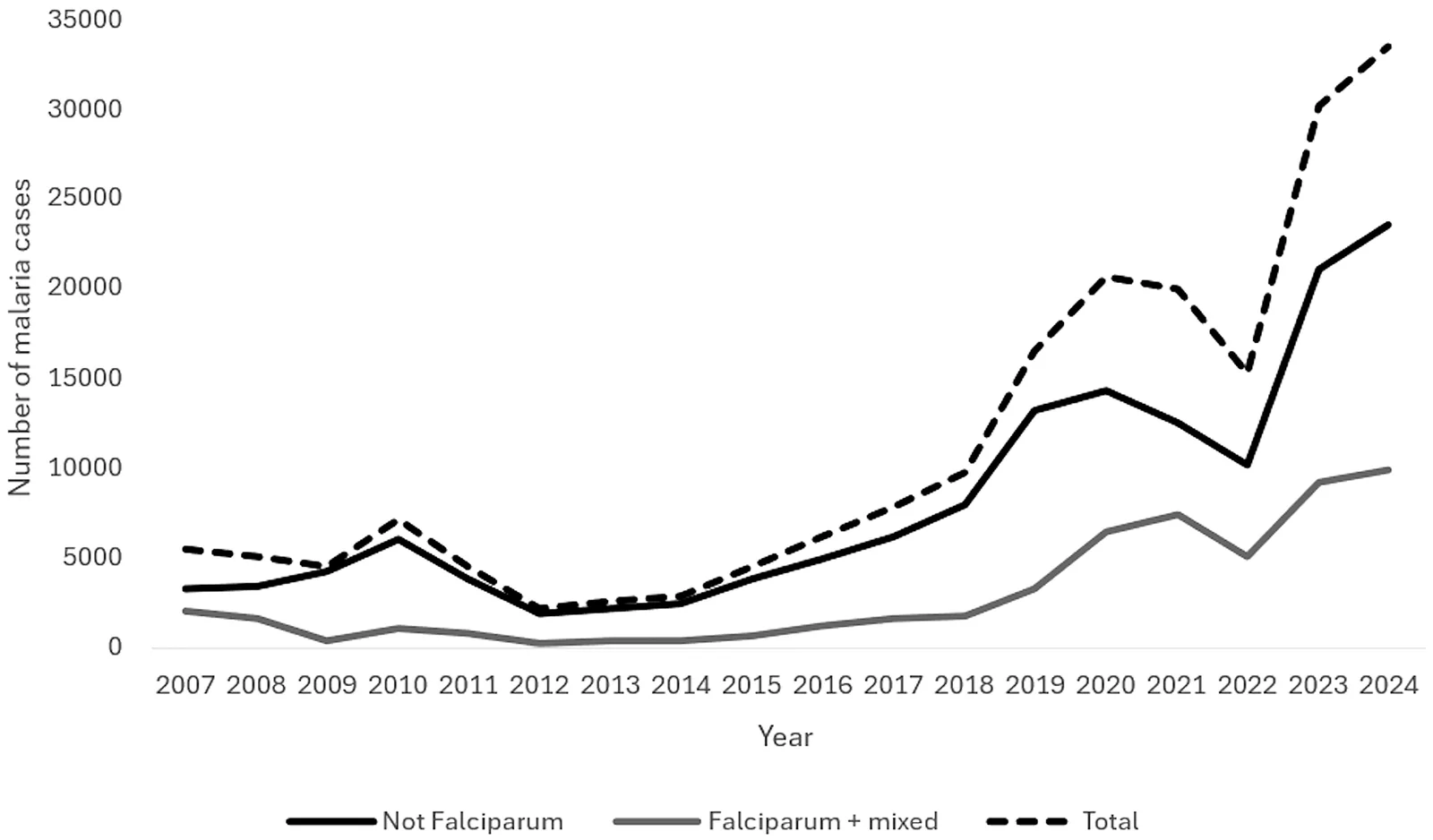
Historical series of malaria cases reported in the Yanomami Indigenous Special Health District, between 2007 and 2024 (Available from: https://public.tableau.com/app/profile/mal.ria.brasil/viz/BoletimMalriaemreasindgenas/Incio, accessed on April 12, 2026). After a period of relative stability, malaria transmission began to increase from 2012 onward, intensifying in subsequent years and reaching approximately 33,000 cases in 2024. This increase occurred across all categories, with non-*falciparum* infections predominating, although *Plasmodium falciparum* and mixed cases also rose substantially.

It is essential to note that the high number of cases registered in 2024 may result exactly from, and not despite, the increased presence and activity of health professionals in the region, leading to more diagnoses, registrations, and case monitoring, which were not previously carried out systematically. However, official data may still be underestimated, mainly due to difficulties in accessing isolated ethnic groups, the semi-nomadic habits of the Yanomami, the operational support provided at the 37 base centres, and logistical and human resource constraints. In fact, in 2024, compared with 2023, there was a 44% increase in the number of diagnostic tests, a 48% increase in the number of tests performed through active case detection, and a 26% increase in the number of tests performed through passive case detection. With expanded access to diagnosis and treatment, the total number of tests conducted in 2024 (260,251) increased by 43.9% compared with 2023 (180,906).^(26)^ However, in 2024 compared with 2023, there was a 42% reduction in malaria-related deaths and a 47% reduction in case fatality rate. In addition, the proportion of positive tests decreased by 24%,^(25)^ which seems to indicate that the increase in the number of cases does not reflect a worsening of the situation, but rather a more intensive effort to fight and control the disease. A study conducted by Barros et al.^(26)^ examined malaria trends before and after the federal government's response in the YIT in 2023 and showed that malaria case detection increased, hospitalisations decreased, and mortality remained stable in the period they analysed.

## MAIN LIMITATIONS FOR MALARIA ELIMINATION IN THE YANOMAMI INDIGENOUS TERRITORY

Despite control measures aimed at eliminating malaria in YIT, significant limitations persist. Disease transmission is not homogeneous throughout the territory, and specific regions exhibit particularly intense *P. falciparum* circulation (Figure).^(27,28)^ Although all regions of the territory are currently at high risk of malaria transmission, this is part of a complex and interrelated set of biological, environmental, operational, and sociocultural factors. The interactions between them create intricate and reinforcing dynamics that sustain high transmission rates, maintain hidden reservoirs of infection, and compromise the effectiveness of control and elimination strategies in YIT. This may be relevant to other remote and vulnerable populations in the Amazon region facing similar ecological and structural challenges.

*Biological determinants* - In YIT, a high prevalence of submicroscopic and oligosymptomatic infections was observed, reaching approximately 80%, as reported by Robortella et al.^(27)^ Because these infections are difficult to detect using conventional diagnostic methods, they can represent a significant portion of the infectious reservoir. Consequently, even in contexts where there is active case finding, a substantial number of these infections may remain untreated, perpetuating malaria transmission in the region. Therefore, submicroscopic and asymptomatic infections constitute a major obstacle to disease control.

*Environmental determinants* - The intensification of mining, coupled with escalating environmental degradation, has transformed the region into a highly conducive environment for malaria transmission. The presence of infected miners, most of whom are asymptomatic, facilitates malaria transmission in Indigenous territories, including the YIT.^(2,17)^ Mining operations not only create numerous water-filled pits but also alter river courses. These modifications increase water surface area and promote stagnation, creating ideal breeding grounds for *Anopheles* mosquitoes, the main vectors of malaria. Additionally, the constant movement of prospectors searching for new mining sites facilitates the dispersal of malaria throughout the territory.^(17,28)^ It is essential to highlight that inadequate treatment or non-adherence to treatment in mining areas has led to the emergence of *Plasmodium* strains resistant to currently available antimalarials, which can spread among the Indigenous population in the YIT, making disease control increasingly difficult. Intertwined environmental and ecological transformations sharply increase the local vector population and sustain continuous malaria transmission dynamics.

Furthermore, YIT harbours a diverse *Anopheles* mosquito community, with previous studies reporting high infection rates among multiple vector species collected locally.^(14,29)^ This entomological diversity likely sustains malaria transmission in a variety of ecological niches, complicating efforts to interrupt the disease cycle.

*Operational and logistical barriers* - The difficulty of reaching isolated ethnic groups and semi-nomadic populations constitutes a substantial barrier to effective malaria control. Isolated ethnic groups and nomadic individuals potentially infected by *Plasmodium* spp. may sustain active transmission whose extent is not fully recognised, since diagnosis, treatment and case notification are all limited in these settings. In general, communities near health centres have greater access to diagnosis and rapid treatment for malaria; therefore, their malaria burden is less severe, and the disease transmission cycle is disrupted in these areas. However, there are more remote communities whose access is limited to missions (temporary relocation of activities to the area for 5-15 days), or even those with virtually no access to healthcare. Such circumstances severely limit the ability to diagnose cases, provide timely treatment, and implement measures to control transmission.

Malaria surveillance in YIT faces additional layers of complexity. Most Indigenous people do not have a fixed name; they have a secret, unpronounceable, or frequently changing name. In addition, referring to the dead (for example, the father or mother) is problematic; recording movements, the grouping and/or regrouping of communities, and the definition of time also pose significant problems.^(4)^ All these variables make it difficult to accurately record and report cases, define likely sites of infection, evaluate recurrences that require changes in therapy, identify potential therapeutic failures, determine the duration of symptoms, and even define malaria treatment.

*Socio-anthropological and cultural determinants* - The concept of disease among Indigenous peoples differs from the biomedical concept, as disease is often related to supernatural events. This difference complicates and delays the diagnosis and treatment of various diseases, including malaria and medication adherence. Delays in the diagnosis and treatment of malaria pose a greater risk of disease transmission, case chronicity, and/or disease severity, with the potential for death, especially in children. Limited awareness of the importance of medication in completely curing malaria can lead to treatment abandonment, mainly after the first days of treatment, when symptoms have resolved. Non-adherence to or incomplete treatment with currently recommended antimalarials increases the risk of therapeutic failure and recurrences in *vivax* malaria, thereby maintaining disease transmission cycles in receptive settings and leading to cases of malaria resistant to available antimalarials.

Implementing effective vector control remains fraught with difficulties. Traditional Indigenous dwellings are often constructed of straw and may lack walls. The customary practice of lighting fires under nets, while culturally important, diminishes the practicality and effectiveness of interventions such as insecticide-treated mosquito nets. Overall, these preventive measures are rarely integrated into daily life, further limiting the impact of standard vector-control approaches in the territory.

These factors do not operate independently. Rather, they interact in complex and reinforcing ways, sustaining high levels of transmission, preserving hidden reservoirs of infection, and ultimately undermining the effectiveness of both control and elimination strategies in YIT.

## SUGGESTED MEASURES TO BE CONSIDERED

It is necessary to consider the goal of malaria elimination in Brazil and to draw up an intensified action plan for the YIT or for other regions with similar characteristics. Considering the persistent challenges to malaria control and elimination in the territory, it is essential to reinforce and implement targeted measures that address the complex interplay of biological, environmental, operational, and sociocultural factors underpinning sustained transmission. Strengthening interventions with an awareness of these multifaceted determinants increases the likelihood of achieving effective and sustainable outcomes in malaria reduction.

Given the current epidemiological context of high malaria transmission and the characteristics of the environment, housing structures, and cultural aspects (limited adherence to the use of insecticide-treated mosquito nets and the lack of a clear definition of "inside" and "outside" in some dwellings without walls), vector-control measures may be less effective when compared with measures based on timely diagnosis and treatment. Strengthening diagnostic, therapeutic, surveillance, and culturally adapted interventions should be prioritised in the territory.

*Biological strategies* - It is necessary to strengthen the diagnostic and therapeutic tools recommended by the MoH, especially the rapid malaria diagnostic test (RDT), to guarantee an accurate and timely diagnosis and treatment, thus interrupting the malaria transmission cycle. RDTs are particularly important within the territory and under current transmission conditions, as they can facilitate diagnostic logistics, are easy to transport, and do not require highly specialised personnel; there is no need for microscopes, quality slide dyes, and/or electrical or solar energy to detect the parasite in thick blood smears. However, microscopic examination of thick blood smears remains the gold standard for malaria diagnosis. Furthermore, the high number of cases in the territory, the logistical challenges of moving teams during missions or transporting large numbers of slides, sometimes far exceeding the World Health Organization (WHO) recommendation (more than 60 slides per day per microscopist), make RDTs an essential tool within the territory. Currently, the National Malaria Prevention and Control Program (PNCM) uses a combined malaria test that detects HRP-II and pLDH from *P. falciparum* and pLDH from *P. vivax*. It offers 100% sensitivity for *P. falciparum* HRP-II, 99.7% for *P. falciparum* pLDH, and 98.2% for *P. vivax*, with a specificity of 99.3%.^(30)^

Antimalarial therapy actions should be strengthened, with emphasis on the treatment of *vivax* malaria, expanding the implementation of tafenoquine, since the number of Indigenous people treated remains very low (approximately 350 individuals) when considering the total number of cases in the territory.^(31)^ Tafenoquine, an anti-hypnozoite agent, shortens *vivax* malaria treatment from 7 to 3 days. This strategy can improve treatment adherence by reducing the duration of the therapeutic regimen and significantly contributing to reducing the *P. vivax* infection reservoir. Therefore, it has the potential to significantly impact disease transmission control.^(31)^ The implementation of paediatric tafenoquine (for individuals weighing more than 10 kg), which was recently incorporated into the Brazilian Unified Health System (SUS), is also particularly relevant, since most malaria cases affect the paediatric population and, in general, the population in the territory weighs less than 36 kg, the minimum weight for the adult tafenoquine presentation under the current protocol.^(31)^

The use of ASMQ as the first-line treatment for *falciparum* malaria and recurrences of *vivax* malaria is essential, as it supports treatment adherence and reduces the need for intensive supervised administration, since dosing is once daily. Given cultural considerations, it is highly recommended that malaria treatment, as well as treatment for other diseases, be assisted by a healthcare professional.

Mass or unified treatment, strategically and supervised in specific malaria-endemic areas, may be considered if a well-defined protocol ensures the feasibility of completing all treatment cycles and patient safety. This strategy can reduce submicroscopic infections, thereby interrupting the malaria transmission cycle. To justify mass or unified treatment, malaria investigations should use a combination of microscopy, rapid diagnostic tests, and molecular techniques to accurately assess the malaria burden. Finally, molecular assessment of potential resistance to currently available antimalarials is recommended, given the challenges of parasite clearance, inadequate treatment adherence due to cultural conditions, and the circulation of miners in the territory, where resistance to these antimalarials has been reported. Assessing resistance may lead to adjustments in currently recommended therapies and help ensure the effectiveness of new interventions.^(32)^

Strengthening the malaria surveillance system, particularly the census, is essential. It is important to emphasise that the census must use technologies that enable rapid and accurate identification of Indigenous people. The technology proposed for incorporation into the census includes fingerprint, palm and/or iris recognition. However, it is necessary to carefully evaluate anthropological considerations and ensure the preservation of Indigenous rights and culture.

The use of smartphone-based surveillance systems can significantly speed up case notification and, at the same time, improve the quality of the data collected. Expanding active case detection to as many villages and communities as possible, with the aim of comprehensive population coverage, is essential to ensure both early diagnosis and timely treatment. Knowing the actual number of malaria cases in the territory, as well as key patient characteristics (age, duration of illness, date of onset, date of symptom return), is essential for guiding more effective malaria control measures and therapeutic decision-making.

It is recommended that individuals seeking care for any reason, as well as their family members, undergo testing to identify asymptomatic or early-stage infections. Furthermore, strengthening the logistical infrastructure, encompassing transportation, supply systems, and the availability of properly trained healthcare professionals, is essential to ensure continuity of care and enable the effective implementation of control measures.

*Sociocultural strategies* - Health education activities focused on the importance of early diagnosis and timely and adequate treatment, with adherence to antimalarial therapy and respect for cultural issues, must be promoted and strengthened. The involvement of Indigenous leaders is strongly recommended, given their role in raising awareness and disseminating knowledge. Educational strategies need to value local knowledge systems and seek to broaden the population's understanding of malaria transmission and treatment. This is expected to promote greater engagement with health services and strengthen adherence to recommended therapies. Such measures should be implemented in a coordinated manner, considering the biological, environmental, operational, and sociocultural determinants that perpetuate malaria transmission in YIT.

The limitations to controlling and eliminating malaria, as well as the proposed strategies to mitigate them, must be considered by public health decision-makers. It is essential to note that some of the recommended measures were included in and implemented through the COE action plan; however, their performance indicators have not yet been adequately reported. These results highlight the urgent need for integrated public health strategies that coordinate disease control, environmental preservation, and culturally sensitive interventions to address malaria in highly vulnerable contexts. Success in meeting this challenge largely depends on coordinating these different dimensions, considering local conditions and community practices.

## IN CONCLUSION

The persistence of malaria in YIT is not merely a health problem, but a symptom of a broader structural, humanitarian, and environmental crisis. The interventions implemented, such as intensified active case finding, expanded diagnosis (including the use of RDTs), and reinforced therapeutic actions (with the introduction of tafenoquine, the use of ASMQ as first-line treatment for *P. falciparum*, and unified or mass treatment), demonstrate the capacity to mitigate immediate damage, as evidenced by the reduction in lethality. However, given the magnitude and persistence of transmission, it is essential to move beyond traditional emergency-response models and advance toward long-term, integrated, and sustainable malaria-elimination strategies. Without coordinated action to address environmental degradation, strengthen the health system, and implement culturally appropriate interventions, malaria elimination in YIT is unlikely to be achieved.

## Data Availability

After publication the data will be available on request from the corresponding author.

## References

[1] MS - Ministério da Saúde (2024). Caracterização da malária em áreas especiais da região amazônica. Boletim Epidemiológico.

[2] Martins-Filho PR, Damascena NP, Araujo APD, Silva MC, Santiago BM, Deitos AR (2023). The devastating impact of illegal mining on indigenous health: a focus on malaria in the Brazilian Amazon. EXCLI J.

[3] Walker RS, Flinn MV, Prall SP, Hamilton MJ (2023). Remote sensing evidence for population growth of isolated indigenous societies in Amazonia. Sci Rep.

[4] Lobo MSC, Cardoso MLM (2023). Lessons from urgent times: the experience of Yanomami health care then and now. Cad Saude Publica.

[5] Grenfell P, Fanello CI, Magris M, Gonçalves J, Metzger WG, Vivas-Martínez S (2008). Anaemia and malaria in Yanomami communities with differing access to healthcare. Trans R Soc Trop Med Hyg.

[6] Albert B, Le Tourneau FM (2007). Ethnogeography and resource use among the Yanomami toward a model of "reticular space". Curr Anthropol.

[7] SESAI - Secretaria de Saúde Indígena População indígena. https://infoms.saude.gov.br/extensions/sesai_pop_indigena/sesai_pop_indigena.html.

[8] Castro MC, Arisco NJ, Diniz CG, Ponmattam J, Peterka C, Basta PC (2025). Mining and malaria in the Brazilian Amazon and in the Yanomami indigenous land. PLoS Negl Trop Dis.

[9] SI - Survival International Yanomami. https://www.survivalinternational.org/peoples/yanomami.

[10] Yanomami Foundation The Yanomami. https://www.yanomamifoundation.org/yanomami.

[11] Toledo AP, Di Benedetto S, Bizawu K (2023). Indígenas Yanomami no Brasil: ordem interna e o sistema interamericano de direitos humanos como indicativo de crise humanitária. Veredas Direito.

[12] Ferguson RB (2015). History, explanation, and war among the Yanomami: a response to Chagnon's Noble Savages. Anthropol Theory.

[13] Rifkin J (1994). Ethnography and ethnocide: a case study of the Yanomami. Dialect Anthropol.

[14] Sánchez-Ribas J, Oliveira-Ferreira J, Gimnig JE, Pereira-Ribeiro C, Santos-Neves MSA, Silva-do-Nascimento TF (2017). Environmental variables associated with anopheline larvae distribution and abundance in Yanomami villages within unaltered areas of the Brazilian Amazon. Parasit Vectors.

[15] Pithan OA, Confalonieri UEC, Morgado AF (1991). A situação de saúde dos índios Yanomámi: diagnóstico a partir da casa do índio de Boa Vista, Roraima, 1987 - 1989. Cad Saude Publica.

[16] Castro MC, Peterka C (2023). Malaria is increasing in Indigenous and artisanal mining areas in the Brazilian Amazon. Nat Med.

[17] de Aguiar Barros J, Granja F, Pequeno P, Marchesini P, Ferreira da Cruz MF (2022). Gold miners augment malaria transmission in indigenous territories of Roraima state, Brazil. Malar J.

[18] Da Luz Scherf E, Viana da Silva MV (2023). Brazil's Yanomami health disaster: addressing the public health emergency requires advancing criminal accountability. Front Public Health.

[19] Paniz-Mondolfi AE, Grillet ME, Tami A, Oliveira-Miranda MA, Delgado Noguera L, Hotez P (2019). Venezuela's upheaval threatens Yanomami. Science.

[20] Athila AR (2022). Epidemias, protagonismos e direitos específicos de saúde: a criação do distrito sanitário Yanomami e a política de saúde indígena no Brasil (1991-2021). Astrolabio. Nueva Época.

[21] MS - Ministério da Saúde COE Yanomami. https://www.gov.br/saude/pt-br/composicao/svsa/coes/coe-yanomami/coe-yanomami.

[22] MS - Ministério da Saúde Portaria GM/MS nº 28, de 20 de janeiro de 2023. Declara Emergência em Saúde Pública de Importância Nacional (ESPIN) em decorrência de desassistência à população Yanomami. https://www.gov.br/saude/pt-br/composicao/svsa/coes/coe-yanomami/publicacoes-tecnicas/decretos-e-portarias.

[23] Ramos AR (1993). O papel político das epidemias: o caso Yanomami. https://saudeindigena.fiocruz.br/handle/bvs/1473.

[24] MS - Ministério da Saúde (2024). Território Yanomami é a primeira região do Brasil a receber medicamento para cura da malária. https://www.gov.br/saude/pt-br/assuntos/noticias/2024/marco/territorio-yanomami-e-a-primeira-regiao-do-brasil-a-receber-medicamento-para-cura-da-malaria.

[25] MS - Ministério da Saúde Missão Yanomami - Informe 07. https://www.gov.br/saude/pt-br/composicao/svsa/coes/coe-yanomami/informes/missao-yanomami-informe-07/view.

[26] de Aguiar Barros J, Granja F, Siqueira PEL, Silva Epifânio I, Coelho RR, Ferreira-da-Cruz MF (2025). What impact did the Brazilian Federal Government's intervention have on malaria cases in the Yanomami Indigenous lands?. Malar J.

[27] Robortella DR, Calvet AA, Amaral LC, Fantin RF, Guimarães LFF, França Dias MH (2020). Prospective assessment of malaria infection in a semi-isolated Amazonian indigenous Yanomami community: transmission heterogeneity and predominance of submicroscopic infection. PLoS One.

[28] Barbieri AF, Santos RO (2025). Human mobility, garimpos and spatiotemporal malaria transmission in the Yanomami Indigenous Territory: a retrospective observational study. Lancet Reg Health Am.

[29] Sánchez-Ribas J, Silva-do-Nascimento TF, Pereira-Ribeiro C, Santos-Neves MSA, Barnwell JW, Lourenço-de-Oliveira R (2025). Bionomics and malaria infection rates in anopheline species in Yanomami villages of the Brazilian Amazon Forest. Malar J.

[30] MS - Ministério da Saúde (2023). Nota Técnica nº 10/2023-CEMA/DEDT/SVSA/MS. Orientações para o uso do teste rápido no diagnóstico de malária. https://www.gov.br/saude/pt-br/centrais-de-conteudo/publicacoes/notas-tecnicas/2023/nota-tecnica-no-10-2023.

[31] MS - Ministério da Saúde (2025). Implementação da tafenoquina nos municípios prioritários do Brasil, 2024. Boletim Epidemiológico.

[32] de Aguiar Barros J, Granja F, de Abreu-Fernandes R, Tavares de Queiroz L, da Silva E Silva D, Citó AC (2024). Investigation of mutations in the *crt-o* and *mdr1* genes of *Plasmodium vivax* for the molecular surveillance of chloroquine resistance in parasites from gold mining areas in Roraima, Brazil. Microorganisms.

