# Supplementary material for: Epidemiology and social factors of malaria in the Yanomami Indigenous Territory: a critical review of the challenges of control and elimination
Source: Mem Inst Oswaldo Cruz. 2026 Oct 9;121:e260033. doi: 10.1590/0074-02760260033 (PMC13652306; doi:10.1590/0074-02760260033)
Supplement: Peer review history

# Reviewer report: FIRST REVIEW ROUND - REVIEWERS' COMMENTS

## REVIEWER #1

Title - Given that a significant portion of the manuscript's analysis focuses on the epidemiological impact of social and environmental determinants in the YIT, a title adjustment is recommended to better reflect this scope.

Suggestion: Epidemiology and Social Drivers of Malaria in the Yanomami Indigenous Territory: A Critical Review of Control and Elimination Challenges.

L79-80 - The text does not clearly specify whether the 2023 increase occurred gradually or abruptly as an epidemic surge. It is important to consider that this rise may be associated with the intensification of diagnostic and reporting efforts during that period.

L88 - The term "malaria situation" is overly generic for an academic review. I suggest replacing it with "epidemiological landscape of the YIT" to enhance technical precision.

Suggestion: The objective of this mini-review is to describe the epidemiological landscape of the YIT...

L99 - The term "acculturation" is considered inappropriate in contemporary Public Health, as it implies a unidirectional loss of culture. I suggest using "social integration" or "varying degrees of contact with surrounding society" to avoid outdated sociopolitical connotations.

L128 - The manuscript's phrasing in certain sections, such as 'major public health problem,' should be refined to reflect a more robust academic register. I suggest: 'Malaria currently represents one of the most critical public health threats within the Yanomami territory, characterized by escalating incidence and profound socio-epidemiological vulnerability.' This adjustment avoids the perception of a literal translation and aligns the text with the technical depth required by the journal.

L158 - To enhance epidemiological precision, I recommend replacing the verb 'rose' with a more descriptive term regarding the transmission dynamics. For instance: 'the annual malaria incidence escalated to [X%]' or 'the transmission rate surged to...'. Using 'escalated' or 'accelerated' better conveys the gravity of the epidemic expansion described in the study.

L160 - The claim that malaria cases were underestimated by 83% in 2022 (based on Castro et al.) is one of the most impactful statements in the manuscript and requires further substantiation. Such a significant gap implies that only 17% of actual cases were diagnosed and treated—a scenario that would suggest a near-total collapse of the healthcare infrastructure in the YIT and a subsequent spike in mortality. The authors should briefly clarify the methodological basis for this estimate (e.g., whether it is based on mathematical modeling or active case-finding) to ensure the findings are credible to the international scientific community.

L195 - Regarding the section 'Main Limitations for Malaria Elimination...', there is a noticeable overlap of distinct variables within the same paragraphs. By merging biological determinants (submicroscopic infections), environmental factors (breeding sites), logistical constraints (distance/access), and anthropological aspects (cultural reluctance) into a single concise paragraph, the authors have inadvertently diluted the individual significance of each driver. Consequently, the manuscript lacks a deeper critical analysis of how each specific determinant impacts the failure of control efforts.

This observation should be taken into account for a comprehensive revision and reorganization of the discussion between lines 200 and 262. I strongly recommend restructuring these paragraphs to address the biological, environmental, and socio-cultural dimensions separately, thereby strengthening the causal arguments and the overall epidemiological reasoning.

I suggest restructuring these paragraphs according to thematic axes to ensure a more rigorous analysis. For example: Biological and Entomological Dynamics Axis

Focus: Silent reservoirs and vector ecology.

Drafting Suggestion: The uneven distribution of malaria in the Yanomami Territory is underpinned by critical biological and environmental factors. The high prevalence of asymptomatic and oligosymptomatic individuals, often harboring submicroscopic infections, creates a persistent and invisible parasite reservoir. This is compounded by an ecological setting highly conducive to Anopheles breeding, with vast, difficult-to-access larval habitats that sustain high vector densities across the region.

Institutional and Logistical Infrastructure Axis

Focus: Healthcare asymmetry (Health Centers vs. Temporary Missions)

Drafting Suggestion: Healthcare infrastructure introduces a second layer of heterogeneity. While communities proximal to health centers benefit from timely diagnosis and treatment—effectively disrupting the transmission cycle—remote populations rely on sporadic 'missions' (5–15 days). This logistical fragility results in a disproportionate malaria burden in the hinterlands, where the absence of continuous surveillance allows outbreaks to remain undetected.

Socio-Anthropological and Cultural Axis

Focus: Cultural barriers and acceptability of treatment.

Drafting Suggestion: Furthermore, control efforts are hampered by complex socio-cultural determinants. In the most isolated areas, historical factors and different health perceptions may lead to a reluctance to engage with non-indigenous health professionals. Without culturally tailored interventions that respect Yanomami social dynamics, standard pharmacological and vector control strategies face significant barriers to effectiveness.

Why this restructuring improves the manuscript:

* Pedagogical Clarity: It presents a logical "ladder" of challenges: the environment (biology), access (logistics), and acceptance (culture), making the complex reality of the YIT more intelligible to the reader.

* Analytical Depth: By isolating these themes, the authors create the necessary space to cite specific literature for each field—ranging from entomology to medical anthropology—thus strengthening the evidence base.

* Epidemiological Rigor: In Public Health, conflating distinct determinants obscures the chain of causality. Categorizing them clarifies that the crisis is not merely a biological phenomenon (the vector), but a consequence of institutional collapse and cultural distancing.

L287 - The manuscript should be updated to include the recent incorporation of the pediatric formulation of tafenoquine by the Brazilian health system. Despite the data cutoff, this represents a major technological advancement for malaria control and should be acknowledged.

L313 - Currently, the text focuses solely on existing barriers; the authors should propose actionable recommendations or interventions. This paragraph requires further development to discuss potential strategic improvements for the control and elimination program.

L384-388 - The graphical analysis requires closer alignment with the data. The chart does not demonstrate the aforementioned decline between 2012 and 2014; conversely, 2012 marks the onset of a steady increase leading to the 2020 peak. I suggest removing the 2025 data points, as they are not analyzed in the text and remain incomplete in SIVEP-Malaria. Furthermore, it is essential to discuss whether the 2022 decline reflects significant underreporting or a genuine decrease in incidence.

## REVIEWER #2

Dear authors,

This is a timely and highly relevant narrative review addressing the humanitarian and epidemiological crisis in the Yanomami Indigenous Territory (YIT). The manuscript successfully integrates environmental, sociocultural, and clinical data to explain the current malaria resurgence. The inclusion of preliminary 2024 data and the discussion of new therapeutic tools (Tafenoquine) make this a significant contribution to the field of tropical medicine and indigenous health.

The abstract is well-structured and provides a concise summary of the historical context, the 2019 escalation, and the current challenges. I recommend including the specific percentage reduction in mortality and case fatality rates observed during the 2024 PHENI (as detailed in the results). This would immediately highlight the positive impact of the emergency interventions to the reader.

The contribution is highly original as it synthesizes recent data from a period of intense public health emergency. Its importance lies in bridging the gap between environmental science (impact of illegal mining) and public health strategy. The focus on submicroscopic infections and sociocultural barriers to treatment adherence offers valuable insights for malaria elimination programs in similar low-resource or cross-border settings.

The manuscript follows a narrative review format. While the sources (official databases and peer-reviewed articles) are reliable, the selection of data appears somewhat directed to support the authors' primary arguments. I suggest the authors briefly clarify the selection criteria or explicitly acknowledge the narrative nature of the review as a limitation to ensure scientific transparency.

In the section "Characterization of the Yanomami Population" (lines 114-118), the tone is somewhat politicized. To maintain the high academic standard of Memórias, I suggest rephrasing the sentence regarding governmental interventions to focus on policy outcomes. For example:

"Changes in environmental and indigenous policies in recent years coincided with an expansion of mining activities... This shift, coupled with gaps in health assistance, has been linked to increased territorial vulnerability."

* This adjustment shifts the focus from political figures to institutional and policy consequences, which is more appropriate for a technical report.

* However it is my take... the author doesn't need to change it if it is intentional.

The discussion of sociocultural barriers (notions of disease, nomadic habits) is excellent and provides a necessary context that is often missing in purely clinical reports.

The references are up to date, including several crucial citations from 2024 and 2025.

## AUTHORS' RESPONSE TO THE REVIEWERS

REVIEWER COMMENTS:

Reviewer: 1

**Title - Given that a significant portion of the manuscript's analysis focuses on the epidemiological impact of social and environmental determinants in the YIT, a title adjustment is recommended to better reflect this scope.**

**Suggestion: Epidemiology and Social Drivers of Malaria in the Yanomami Indigenous Territory: A Critical Review of Control and Elimination Challenges.**

*Response:* The title has been changed. In the revised text, it appears as: "EPIDEMIOLOGY AND SOCIAL FACTORS OF MALARIA IN THE YANOMAMI INDIGENOUS TERRITORY IN BRAZIL: A CRITICAL REVIEW OF THE CHALLENGES AND POTENTIAL LIMITATIONS FOR CONTROL AND ELIMINATION"

**L79-80 - The text does not clearly specify whether the 2023 increase occurred gradually or abruptly as an epidemic surge.**

**It is important to consider that this rise may be associated with the intensification of diagnostic and reporting efforts during that period.**

*Response:* We acknowledge that, in the original version of the text, it was not sufficiently clear whether the increase observed in 2023 was the result of a gradual process or corresponded to an abrupt epidemic outbreak. For this reason, we made adjustments to the manuscript to clarify that the rise in malaria cases in that year should be interpreted with caution. Instead of indicating a sudden outbreak, the recorded growth appears to be related to both the persistent intensification of transmission and the gradual advancement in surveillance, diagnosis, and notification strategies implemented during the period, resulting in increased case detection. A passage was inserted explicitly explaining this interpretative caution: "Interpreting this increase demands caution. Rather than reflecting the onset of a sudden epidemic, the data more likely point to a steady, ongoing intensification of malaria transmission..." ...expanded surveillance efforts, improved diagnostic access, and, consequently, more thorough case documentation..."

Furthermore, in the epidemiological section, it was emphasized that the higher number of cases in 2024 may also result from the increased presence of teams and diagnostic capacity: "It is essential to note that the high number of cases registered in 2024 may result exactly from... the increased presence and activity of health professionals in the region, leading to more diagnoses, registrations, and case monitoring..."

**L88 - The term "malaria situation" is overly generic for an academic review. I suggest replacing it with "epidemiological landscape of the YIT" to enhance technical precision.**

**Suggestion: The objective of this mini-review is to describe the epidemiological landscape of the YIT ...**

*Response:* It was changed, as suggested. The objective now uses the more technical formulation: "This mini-review aims to describe the epidemiological landscape in YIT..."

**L99 - The term "acculturation" is considered inappropriate in contemporary Public Health, as it implies a unidirectional loss of culture. I suggest using "social integration" or "varying degrees of contact with surrounding society" to avoid outdated sociopolitical connotations.**

*Response:* We agree that the term "acculturation" can carry a reductionist connotation. We have revised the manuscript to replace the term with more appropriate expressions. In the section describing the population, the idea was reformulated to: "some groups remain isolated, while others demonstrate varying degrees of contact with society at large."

**L128 - The manuscript's phrasing in certain sections, such as 'major public health problem,' should be refined to reflect a more robust academic register. I suggest: 'Malaria currently represents one of the most critical public health threats within the Yanomami territory, characterized by escalating incidence and profound socio-epidemiological vulnerability.' This adjustment avoids the perception of a literal translation and aligns the text with the technical depth required by the journal.**

*Response:* The passage has been revised and the phrasing was changed, as suggested. The phrase was strengthened and came to say: "Malaria currently represents one of the most critical public health threats in the Yanomami territory, characterized by increasing incidence and profound socio-epidemiological vulnerability."

**L158 - To enhance epidemiological precision, I recommend replacing the verb 'rose' with a more descriptive term regarding the transmission dynamics. For instance: 'the annual malaria incidence escalated to [X%]' or 'the transmission rate surged to...'. Using 'escalated' or 'accelerated' better conveys the gravity of the epidemic expansion described in the study.**

*Response:* We agree that the use of terms like "increased" can limit the interpretation of the magnitude. The passage now describes the change more analytically: "the average annual growth rate of malaria rose to 30.2%... more than tripling the rate of disease spread" and immediately afterwards: "translating into a marked acceleration in malaria transmission."

**L160 - The claim that malaria cases were underestimated by 83% in 2022 (based on Castro et al.) is one of the most impactful statements in the manuscript and requires further substantiation. Such a significant gap implies that only 17% of actual cases were diagnosed and treated—a scenario that would suggest a near-total collapse of the healthcare infrastructure in the YIT and a subsequent spike in mortality. The authors should briefly clarify the methodological basis for this estimate (e.g., whether it is based on mathematical modeling or active case-finding) to ensure the findings are credible to the international scientific community.**

*Response:* We agree that the 83% underreporting estimate for 2022 represents a finding of great magnitude. We revised the manuscript to clarify that this estimate, as described by Castro et al. The methodological explanation and the interpretative caveat were included: "Some projections suggest that underreporting of malaria cases in 2022 may have reached rates as high as 83%..." "This was based on models presented by Castro et al. and on results from integrating epidemiological data and environmental indicators..." "However, it is crucial to interpret this estimate with caution, as it relies on inferences... rather than on data derived from the active and direct detection of cases."

**L195 - Regarding the section 'Main Limitations for Malaria Elimination...', there is a noticeable overlap of distinct variables within the same paragraphs. By merging biological determinants (submicroscopic infections), environmental factors (breeding sites), logistical constraints (distance/access), and anthropological aspects (cultural reluctance) into a single concise paragraph, the authors have inadvertently diluted the individual significance of each driver. Consequently, the manuscript lacks a deeper critical analysis of how each specific determinant impacts the failure of control efforts.**

This observation should be taken into account for a comprehensive revision and reorganization of the discussion between lines 200 and 262. I strongly recommend restructuring these paragraphs to address the biological, environmental, and socio-cultural dimensions separately, thereby strengthening the causal arguments and the overall epidemiological reasoning.

I suggest restructuring these paragraphs according to thematic axes to ensure a more rigorous analysis. For example:

Biological and Entomological Dynamics Axis

Focus: Silent reservoirs and vector ecology.

Drafting Suggestion: The uneven distribution of malaria in the Yanomami Territory is underpinned by critical biological and environmental factors. The high prevalence of asymptomatic and oligosymptomatic individuals, often harboring submicroscopic infections, creates a persistent and invisible parasite reservoir. This is compounded by an ecological setting highly conducive to Anopheles breeding, with vast, difficult-to-access larval habitats that sustain high vector densities across the region.

Institutional and Logistical Infrastructure Axis

Focus: Healthcare asymmetry (Health Centers vs. Temporary Missions)

Drafting Suggestion: Healthcare infrastructure introduces a second layer of heterogeneity. While communities proximal to health centers benefit from timely diagnosis and treatment—effectively disrupting the transmission cycle—remote populations rely on sporadic 'missions' (5–15 days). This logistical fragility results in a disproportionate malaria burden in the hinterlands, where the absence of continuous surveillance allows outbreaks to remain undetected.

Socio-Anthropological and Cultural Axis

Focus: Cultural barriers and acceptability of treatment.

Drafting Suggestion: Furthermore, control efforts are hampered by complex socio-cultural determinants. In the most isolated areas, historical factors and different health perceptions may lead to a reluctance to engage with non-indigenous health professionals. Without culturally tailored interventions that respect Yanomami social dynamics, standard pharmacological and vector control strategies face significant barriers to effectiveness.

Why this restructuring improves the manuscript:

* Pedagogical Clarity: It presents a logical "ladder" of challenges: the environment (biology), access (logistics), and acceptance (culture), making the complex reality of the YIT more intelligible to the reader.

* Analytical Depth: By isolating these themes, the authors create the necessary space to cite specific literature for each field—ranging from entomology to medical anthropology—thus strengthening the evidence base.

* Epidemiological Rigor: In Public Health, conflating distinct determinants obscures the chain of causality. Categorizing them clarifies that the crisis is not merely a biological phenomenon (the vector), but a consequence of institutional collapse and cultural distancing.

*Response:* We agree that the previous version of the "Main Limitations for Malaria Elimination" section had several different determinants in concentrated in a paragraph, decreasing the importance of each of them. The section was clearly divided into subtopics, with new thematic structures: Biological determinants, Environmental determinants, Operational and logistical barriers and Socio-anthropological and cultural determinants An integrative summary sentence was also inserted: "These factors do not operate independently. Rather, they interact in complex and reinforcing ways..."

**L287 - The manuscript should be updated to include the recent incorporation of the pediatric formulation of tafenoquine by the Brazilian health system. Despite the data cutoff, this represents a major technological advancement for malaria control and should be acknowledged.**

*Response:* It was already there, but with little impact. The text has been reformulated. Specific emphasis was added to the pediatric formulation: "The implementation of pediatric tafenoquine (for individuals weighing more than 10 kg), which was recently incorporated into the Brazilian Unified Health System (SUS), is also particularly relevant..." "...since most malaria cases affect the pediatric population..."

**L313 - Currently, the text focuses solely on existing barriers; the authors should propose actionable recommendations or interventions. This paragraph requires further development to discuss potential strategic improvements for the control and elimination program.**

*Response:* We revised the guidelines to incorporate strategic recommendations aligned with the key determinants of transmission identified in the study. Specifically, we included proposals to strengthen diagnosis and treatment, intensify active surveillance, integrate with environmental actions, and develop culturally adapted approaches to improve treatment adherence. The section "SUGGESTED MEASURES TO BE CONSIDERED" was expanded with concrete recommendations, including: strengthening RDTs and timely diagnosis; expanding tafenoquine and ASMQ use; the possibility of mass or unified treatment under a defined protocol; molecular surveillance of resistance; strengthening the census and smartphone-based surveillance; increasing active case finding; culturally adapted health education; and the need to structurally address illegal mining

**L384-388 - The graphical analysis requires closer alignment with the data. The chart does not demonstrate the aforementioned decline between 2012 and 2014; conversely, 2012 marks the onset of a steady increase leading to the 2020 peak. I suggest removing the 2025 data points, as they are not analyzed in the text and remain incomplete in SIVEP-Malaria.**

*Response:* We revised the time series description to more accurately reflect the observed pattern, removing the reference to a decline between 2012 and 2014 and highlighting that 2012 marks the beginning of a progressive increase in cases. In the legend, the time series pattern was rewritten as: "After a period of relative stability... malaria transmission began to increase from 2012 onward..." And in the data block attached at the end of the file, 2025 still appears in the raw table, but the reviewer's comment was incorporated primarily into the analytical description of the series, avoiding interpreting it as a consolidated trend.

Reviewer: 2

Dear authors,

This is a timely and highly relevant narrative review addressing the humanitarian and epidemiological crisis in the Yanomami Indigenous Territory (YIT). The manuscript successfully integrates environmental, sociocultural, and clinical data to explain the current malaria resurgence. The inclusion of preliminary 2024 data and the discussion of new therapeutic tools (Tafenoquine) make this a significant contribution to the field of tropical medicine and indigenous health.

The abstract is well-structured and provides a concise summary of the historical context, the 2019 escalation, and the current challenges. I recommend including the specific percentage reduction in mortality and case fatality rates observed during the 2024 PHENI (as detailed in the results). This would immediately highlight the positive impact of the emergency interventions to the reader.

*Response:* We recognize that presenting quantitative impact data in the abstract significantly clarifies and makes the results more relevant. Following this guidance, we have revised the abstract to include the observed reductions: 42% in mortality and 47% in the case fatality rate. In the abstract, the numbers were inserted: "a 42% decrease in deaths and a 47% decline in the case-fatality rate"

The contribution is highly original as it synthesizes recent data from a period of intense public health emergency. Its importance lies in bridging the gap between environmental science (impact of illegal mining) and public health strategy. The focus on submicroscopic infections and sociocultural barriers to treatment adherence offers valuable insights for malaria elimination programs in similar low-resource or cross-border settings.

The manuscript follows a narrative review format. While the sources (official databases and peer-reviewed articles) are reliable, the selection of data appears somewhat directed to support the authors' primary arguments. I suggest the authors briefly clarify the selection criteria or explicitly acknowledge the narrative nature of the review as a limitation to ensure scientific transparency.

*Response:* We reviewed the manuscript to briefly clarify the criteria for selecting sources and the cautions regarding the potential biases of narrative texts. A methodological paragraph was added that was not previously so explicit: "This narrative mini-review was based on secondary data obtained from peer-reviewed scientific articles, official surveillance databases, epidemiological bulletins, and institutional reports..." "A systematic search protocol was not adopted, which may introduce some selection bias."

In the section "Characterization of the Yanomami Population" (lines 114-118), the tone is somewhat politicized. To maintain the high academic standard of Memórias, I suggest rephrasing the sentence regarding governmental interventions to focus on policy outcomes. For example:

"Changes in environmental and indigenous policies in recent years coincided with an expansion of mining activities... This shift, coupled with gaps in health assistance, has been linked to increased territorial vulnerability."

* This adjustment shifts the focus from political figures to institutional and policy consequences, which is more appropriate for a technical report.

* However it is my take... the author doesn't need to change it if it is intentional.

*Response:* We revised the passage to adopt a more analytical and results-oriented tone, as suggested. The text is now more institutional and less personalistic: "In recent years, the pace of mining activity has intensified further, amid changes in environmental and indigenous policies." "Adding to this situation is the persistence of obstacles to access to healthcare and basic services..." "...these factors contributed to one of the worst public health emergencies associated with Indigenous peoples in Brazil..."

The discussion of sociocultural barriers (notions of disease, nomadic habits) is excellent and provides a necessary context that is often missing in purely clinical reports.

The references are up to date, including several crucial citations from 2024 and 2025.

Declaration: ChatGPT (OpenAI, Inc.), and Grammarly (Grammarly Inc.) were used to assist with linguistic review and to improve clarity, readability, and style. All scientific content, analyses, and interpretations are the responsibility of the authors.

# Reviewer report: REVIEWERS' COMMENTS

## REVIEWER #1

The abstract is consistent with the manuscript's content. The research proposal is original and presents relevant findings, considering the epidemiological importance of malaria in the Amazon region and among Indigenous populations, particularly within the Yanomami ethnic group, which continues to face challenges in malaria control within its territory. The methodology is appropriate to achieve the proposed objectives. The discussion, references, and figure are adequate. No tables are included in the manuscript.

## REVIEWER #2

No comments.
